# PSYCHOSOCIAL PHENOTYPES OF OLDER ADULTS WITH PAIN AND THEIR ASSOCIATED CLINICAL OUTCOMES

**DOI:** 10.1093/geroni/igad104.3541

**Published:** 2023-12-21

**Authors:** Ashleigh Holmes, Weijun Wang, Yu-Ping Chang

**Affiliations:** University at Buffalo, The State University of New York, Buffalo, New York, United States; University at Buffalo, The State University of New York, Buffalo, New York, United States; University at Buffalo, The State University of New York, Buffalo, New York, United States

## Abstract

Older adults experience higher rates of pain than any other age group, with a domino effect on quality of life and well-being. Existing literature reveals associations between pain and adverse psychosocial, physical, and cognitive outcomes, with psychosocial variables linking all other aspects of well-being in older adults. Therefore, a comprehensive understanding of the associations of psychosocial variables with clinical outcomes in older adults with pain is needed. Grounded in the Biopsychosocial Model of Chronic Pain for Older Adults, we analyzed cross-sectional 2021 data from the nationally representative survey conducted by the National Health and Aging Trends Study. We performed latent class analysis to identify psychosocial clusters (including anxiety, depression, affect, self-realization, resilience, and social participation) of older adults with pain and then used multiple regression analyses to compare pain characteristics, physical health, and cognition among different clusters. We identified four clusters: “Intermediate Psychosocial Phenotype,” “Favorable Psychosocial Phenotype,” “Adverse Psychosocial Phenotype,” and “Compensated Psychosocial Adversity.” Clusters with more psychosocial adversity had worse pain, physical, and cognitive outcomes while controlling for race/ethnicity, gender, and age. Increased self-realization and resilience were seen in clusters with better clinical outcomes. Our findings provide important information for precision pain management interventions for older adults based on their psychosocial phenotypes. Further research should analyze longitudinal trajectories of psychosocial phenotypes as older adults’ pain experience progresses. Clinically, pain treatment decisions should include a comprehensive evaluation of psychosocial factors, with associated health policy initiatives aimed at assessing differences in care utilization and cost among older adults based on phenotype.

